# Type III interferon, age and IFNL gene single nucleotide polymorphisms determine the characteristics of H1N1 influenza infection

**DOI:** 10.3389/fimmu.2025.1592841

**Published:** 2025-05-14

**Authors:** Wenbo Zhu, Shao Wang, Chenchen Guan, Shuangquan Liu, Hongbo Zhang

**Affiliations:** ^1^ The First Affiliated Hospital, Clinical Medical Research Center, Hengyang Medical School, University of South China, Hengyang, Hunan, China; ^2^ Institute of Animal Husbandry and Veterinary Medicine, Fujian Academy of Agriculture Science, Fuzhou, China; ^3^ The First Affiliated Hospital, Clinical Laboratory, Hengyang Medical School, University of South China, Hengyang, Hunan, China; ^4^ Department of Pediatrics, University of Pittsburgh School of Medicine, Pittsburgh, PA, United States

**Keywords:** type III interferon, single nucleotide polymorphisms, age, obesity, influenza infection

## Abstract

**Background:**

Host factors, such as innate immune response, genetic polymorphisms, age, and body weight are important determinants of susceptibility, severity, and responsiveness to treatment of influenza disease. However, the molecular mechanisms underlying these clinical associations remain poorly characterized, particularly regarding IFN-λ-mediated antiviral responses.

**Methods:**

Wild-type mice and IL-28B^-/-^ mice were used to systematically investigate the antiviral and anti-inflammatory functions of IL-29 or IL-28, respectively. Plaque assay and DNA genotyping were conducted to determine the correlations between IFN-λ polymorphisms and H1N1 infection outcomes. ELISA, Real-time PCR and luciferase reporter assays were carried out to explore the mechanism.

**Results:**

IFN-λ plays an important antiviral and immunoprotective role in H1N1 infection. Specifically, IL-29 and IL-28 exhibit important dual antiviral and anti-inflammaroty roles. Age factor also affects H1N1 clearance and therapeutic responsiveness. Human alveolar epithelial cells (AECs) from young donors support higher H1N1 replication and weak response to antiviral treatment with IL-29. Rs12979860 (IL-28 C/T), rs8099917 (IL-28 T/G) and rs30461 (IL-29 A/G), the three identified single nucleotide polymorphisms (SNPs) in IFNL genes, are also associated with H1N1 outcomes. AECs from rs12979860TT and rs8099917GG donors exhibit higher H1N1 replication and nonresponsiveness to IL-29 antiviral therapy. AECs from rs12979860 TT donors also produce lower levels of IFN and exhibit inhibited promoter activity of IL-29 in response to H1N1 infection. In addition, increased allele frequencies of rs12979860 T and rs8099917 G were associated with higher BMI, another important factor influencing H1N1 susceptibility.

**Conclusions:**

This is the first study to comprehensively explore the impact of host factors, especially IFN-λ polymorphisms, on H1N1 virus infection. Further elucidation of the underlying mechanisms may help to develop novel prevention and treatment strategies for influenza virus infection.

## Introduction

The influenza A/H1N1 virus cause seasonal flu and occasionally pandemics. One of the H1N1 influenza virus that emerged in 2009 is known as H1N1pdm09, which is a strain that have crossed the specie barriers from swine to human, leading to serious outbreaks and global pandemic (1). Since 2009, H1N1pdm09 has gradually replaced the old lineages and is circulating in the human populations every year (2). However, it still has the potential to cause the next pandemic. Unlike seasonal influenza viruses targeting the upper respiratory tract, H1N1pdm09 virus-induced lower respiratory tract infections often cause damage to distal lung cells, leading to severe pneumonia (3, 4). Furthermore, H1N1pdm09 strain is associated with a higher attack rate in young individuals. 80% of deaths associated with the H1N1pdm09 virus occur in people under 65 years old. This is very different from the typical seasonal influenza pandemic, during which an estimated 80% of deaths occur in people aged 65 and above (5). In addition, pregnant women and obese patients infected with H1N1 are thought to have an increased risk of serious illness and adverse fatal consequences (6, 7). However, although the disease characteristics of H1N1 virus are clear, the underlying mechanisms and the influence of host factors on the virus have not been fully defined.

Interferons (IFN) are cytokines that are secreted by host cells in response to virus infection and can trigger transcriptional activation of IFN-stimulated genes (ISGs), which in turn exert an antiviral and immune regulatory role. There are three types of IFNs. Members of type I IFN family include IFN-α (with 12 isoforms in human and at least 14 isoforms in mouse), IFN-β, IFN-κ, IFN-ϵ and limitin, which can be produced by various cell types and bind to a common heterodimeric IFN-α/β receptor (IFNAR1/2) that are ubiquitously expressed for signaling (8). IFN-γ is the only type II interferon that is produced primarily by activated NK cells and T cells. It signals through the IFNγ receptors (IFNGR) and is involved in manipulating the acute viral infections, contributing to adaptive immune modulation (9). The third type of IFNs, also known as IFNλs, consists of IFNλ1(IL-29), IFNλ2(IL-28A), IFNλ3(IL-28B), and the most recently discovered IFNλ4. These type III interferons bind to a distinct heterodimeric receptor complex composed of IL10Rβ and IFNLR1, which are expressed preferentially on epithelial cells and few immune cells, mainly neutrophils and dendritic cells (DCs) (10). Studies have consistently confirmed that both type I and type III interferons exert antiviral activity against influenza virus infections (11–13). The physiological role of IFN-γ, the only type II interferon, in influenza virus infection remains unclear, although increased IFN-γ production has been observed in the respiratory tract during influenza infection (14). Most studies have indicated that IFN-γ seems to have no protective function against influenza infection (15, 16). However, other reports suggested that IFN-γ deficiency may lead to increased viral burden or mortality (17, 18). Majority of the antiviral actions of type I and type III IFN are exerted through the expression of interferon-stimulated gene (ISGs). Moreover, IFNλs play an important role in the antiviral defense of the local mucosal barriers and exhibit less pro-inflammatory activities than type I interferons (19). Despite their well-known -antiviral effect at mucosal surface, roles of IFN-λ in viral defense upon H1N1 infection are largely unknown, and the host factors that determine IFN-λ responses in H1N1 infected individuals has not been comprehensively investigated.

Genetic factors are key determinants of viral clearance, treatment response and disease outcome. Single nucleotide polymorphisms (SNPs) located within the IFNL genes, such as rs12979860 (IL-28 C/T), rs8099917 (IL-28 T/G) and rs30461 (IL-29 A/G) have been identified by several independent genome-wide studies (20). Studies have shown that the SNPs rs12979860 and rs8099917 are associated with HCV infection outcomes and their response to antiviral treatment. Among them, rs12979860 CC and rs8099917 TT are favorable genotypes, which may tend to induce spontaneous clearance of HCV virus and better response to IFN therapy (21, 22). SNP rs8099917 was associated with the severity of respiratory syncytial virus bronchiolitis in hospitalized pediatric patients (23). Nonetheless, the biological effects of these functional polymorphisms remain largely unknown. And to date, there are no studies on the relationship between genetic polymorphisms of these IFNL genes with the susceptibility, outcomes and therapeutic responsiveness to H1N1 infection.

In this study, we determined the association of host factors, such as type III interferon response, age factors, and SNPs of the IFNL gene, with the hallmarks of H1N1 virus infection, such as H1N1 virus infectivity and replication capacity, innate immune response, and responsiveness to IL-29 therapy. Additionally, our study explored the potential association between genetic polymorphisms of IFNL genes with H1N1 infection and indicated that rs12979860 TT and rs8099917 GG may be the unfavorable genotypes for host in response to H1N1 virus infection, with increased viral replication, reduced viral clearance, reduced response to IL-29 therapy, and increased disease severity. Our study highlighted the IFN-λ signaling axis as a potential therapeutic target and revealed the potential mechanisms of host factors, especially SNPs of IFNL gene, in influencing the characteristics of H1N1 infection, providing a basis for guiding the development of a more effective anti-influenza treatment regimen.

## Materials and methods

### Donors of human lungs

Lungs from de-identified human donors not suitable for transplantation and donation for medical research were obtained through the International Institute for Medical Development (Edison, New Jersey) and the National Disease Research Exchange (Philadelphia, Pennsylvania). The Committee for Oversight of Research and Clinical Training Involving Decedents and University of Pittsburgh Institutional Review Board approved use of the human tissues.

### Isolation and culture of human AECs

Human alveolar type II cells were isolated from lungs of de-identified healthy donors using a previously described method (24, 25). Briefly, the donated lungs were perfused, lavaged and digested with elastase (12 units/ml; Roche Diagnostics, Indianapolis, USA) and incubated at 37°C for 50min. After the lungs were minced, a suspension of lung cells was filtered through a series of filters, and the erythrocytes inside were lysed in lysis buffer. The AECs were purified by discontinuous density gradient centrifugation at densities of 1.080 and 1.040 and EpCAM microbeads (Miltenyi Biotec Inc., San Diego, CA) positive selection. The isolated type II cells were stained by flow cytometry with pro-SP-C and ATII-280 (ATII-specific marker) to assess their purity (26). Freshly isolated ATII cells were -resuspended in DMEM containing 10% FBS and plated onto transwell inserts (Corning, USA) coated with a mixture of rat tail collagen and matrix gel (BD Biosciences, Bedford, MA). After 48 hours for adherence, the medium was changed to DMEM containing 5% FBS. The AECs were incubated for another 6 days prior to infection with influenza virus.

### Virus preparation

Cal04, a 2009 pandemic H1N1 (H1N1 pdm09) virus strain was provided by courtesy of NIAID BEI Biological Resources. In contrast, NY1682 virus, another H1N1 pdm09 virus strain, was isolated from a patient in New York in April 2009. In addition, the influenza A/PR/8/34, a lab-adapted H1N1 virus, was kindly provided by Dr. K. Hartshorn of Boston University. All viruses were passaged and titrated in MDCK cells as mentioned previously (27). In brief, the purified viruses were successively diluted with DMEM containing 1μg/ml TPCK trypsin (Sigma-Aldrich, St. Louis, MO) and then inoculated into three replicate wells lined with nearly fused MDCK cells. One hour after inoculation, the inoculants were removed and the cells were re-covered with DMEM containing 5% FBS and 0.8% SeaKem LE agarose (Cambrex, Rockland, ME). After incubation at 37°C for 72 hours, plaques were stained and counted with agarose overlayed medium supplemented with 10% neutral red (Sigma-Aldrich, St. Louis, MO).

### Influenza virus infection of AEC cells

Human AECs were cultured prior to infection with viruses to obtain differentiated phenotypes. On Day 6 of culture, the cells were washed with DMEM and inoculated with H1N1 viruses at a multiplicity of infection (MOI) of 1. After incubation at 37°C for 1 hour, the cells were washed twice and re-covered with fresh media. Cell culture supernatants were collected 24 hours after viral infection for viral titration and cytokine analysis (IL-29 and IP-10). In addition, total RNA was harvested 24 hours after viral infection to assess IFN and IFN receptor expression by real-time RT-PCR.

### Virus replication analysis

Virus replication can be evaluated by titration using plaque assay as previously described (27, 28).

### IFN treatment of AEC cells

Human IFN-β (100IU/ml; Gibco, USA) and IL-29 (10ng/ml; R&D Systems, USA) were used to treat human AEC cells. IFN-β or IL-29 was added 1 hour prior to virus inoculation, and IFN-β or IL-29 treatment was continued until 24 hours after virus infection, then cell supernatants were harvested to measure virus titers by plaque assay.

### Quantitative real-time RT-PCR

Quantitative real-time RT-PCR was conducted to determine the mRNA expression of IFN and IFN receptors. Total RNA from AECs with rs12979860 CC/CT or rs12979860 TT was extracted by TRIzol reagents (Invitrogen, USA) and RNeasy Mini Kits (Qiagen, Germany). cDNA was generated by qScript cDNA Synthesis kits (Quanta Bioscience, USA) using 1 microgram DNase-treated RNA as template. Real-time PCR was run in an Applied Biosystems 7900HT real-time PCR System (Life Technologies, USA) by using TB Green™ Premix Ex Taq™ II (Takara, Japan). A two-step PCR program was used for amplification: hot start at 95°C for 15min, followed by 42 cycles at 95°C for 20s and 60°C for 1min. Relative mRNA level was quantified using the 2^-△Ct^ method and standardized to the level of GAPDH.

### Enzyme-linked immunosorbent assay

AEC cells were infected or uninfected with Cal04 or NY1682 or PR8 virus, and the supernatants were collected 24 hours after virus infection. The secretion of IL-29 and IP-10 were measured by ELISA kits following the manufacturer’s instructions. Kit for IL-29 was purchased from ELISA Tech (ELISA Tech, USA) and kit for IP-10 was purchased from Invitrogen (Invitrogen, USA).

### DNA extraction and genotyping

Genomic DNA was extracted from AECs isolated from different lung donors using the QIAamp DSP DNA Mini Kit (Qiagen, Germany) according to the manual instructions. Determination of the rs12979860 (IL28 C/T), rs8099917 (IL28 G/T) and rs30461 (IL29 T/C) gene polymorphisms was performed by the PCR-based restriction fragment length polymorphism (PCR-RFLP) method as described previously (20).

### Luciferase reporter assay

Notably, IL-28A and IL-29 genes are located on the positive DNA strand of chromosome 19, while IL-28B gene is located on the negative DNA strand of this chromosome. The rs12979860 T/C polymorphism is located upstream of the promoter region of all of these genes and thus could in principle affect all three IFN-λ genes (29). Therefore, the impact of SNP rs12979860 on the promoter activity of IL-29 were measured using a luciferase reporter assay. Briefly, genomic DNA was extracted from healthy adult lung donors (#40: donor 40 with rs12979860 CC; #46: donor 46 with rs12979860 CT; #64: donor 64 with rs12979860 TT) using QIAamp DSP DNA Mini kit (Qiagen, Germany). IL-29’s promoter was amplified using primers: forward, 5’-TTTATAAGATCTTTAAACCAATGGCAGAAGCTCC-3’; reverse, 5’- ATATATGGTACCGGCTAAATCGCAACTGCTTCCCCAG-3’. Three reporter plasmids were constructed by inserting promoters with CC, CT or TT genotype separately into the polyclonal sites of the pGL3 basic vectors to drive the firefly luciferase gene expression (Promega, USA). A549 cell lines were transfected with each reporter plasmid DNA by using Lipofectamine 3000 (ThermoFisher Scientific, USA). Briefly, cells in 12-well plates at a density of 3×10^5^ cells/well were transfected with 750 ng of pGL3 (IL-29prom-CC or IL-29prom-CT or IL-29prom-TT) plasmids and 100ng of Renilla luciferase reporter plasmids. All transfections were carried out in triplicate. At 24 hours post transfection, cells were inoculated with Cal04 or PR8 virus at MOI of 1. Luciferase activity was assessed by using dual-luciferase reporter assay kit (Promega, USA) according to the manufacturer’s instructions.

### Murine infections

Wild-type C57BL/6 mice and IL-28B^-/-^(IFNλ3^-/-^) with the C57BL/6NTac background (Ifnl3^tm1.1(KOMP)Vlcg^ mice) were purchased from Jackson Laboratory (Bar Harbor, ME). The wild-type C57BL/6 mice were co-housed and bred with sex-matched IL-28B^-/-^ mice, and the resulting heterozygous F1 offspring were further bred to each other to obtain F2 generations with knockout or wild-type mice. The wild-type C57BL/6 mice, heterozygous IL-28B^+/-^ mice and homozygous IL-28B^-/-^ mice were used for experiments at six to eight weeks of age. All mice were housed under pathogen-free conditions at the Children’s Hospital of Pittsburgh, University of Pittsburgh of Medical Center. All animal studies were conducted on age- and sex-matched mice and approved by the University of Pittsburgh’s Institutional Animal Care and Use Committee.

For influenza virus infection, wild-type C57BL/6 mice were inoculated with 50 μl (10^10^ PFU) each of IL-29-expressing adenovirus or IL-28-expressing adenovirus or control GFP-expressing adenovirus via the intranasal route. Two days after adenovirus infection, mice were challenged with 100 pfu of PR8 virus. On days 3 and 7 after PR8 virus infection, mice were harvested. The weight of each mouse was weighed and recorded, and the lungs were lavaged with 1 ml of sterile PBS without protease inhibitors, the PR8 virus titers in bronchoalveolar lavage fluid (BALF) was measured by plaque assays.

For bacterial infection, wild-type C57BL/6 mice, heterozygous IL-28B^+/-^ mice and homozygous IL-28B^-/-^ mice were inoculated with 100 pfu of PR8 virus and then challenged with 5×10^7^ CFU -*streptococus pneumoniae* in 50 μl PBS per mouse and harvested 48 hours later. The weight of each mouse was weighed and recorded. The lungs were lavaged with 1 ml of sterile PBS without protease inhibitors. The infiltrated cells in BALF were pelleted by centrifugation and resuspended in 500μl of PBS. The total cell count was then determined using a hemocytometer. The bacterial burden and virus burden in the lungs was determined by the previously described method (30).

### Statistical analysis

GraphPad Prism version 6.0 (GraphPad Software, USA) was operated for statistical analysis. Paired Student’s t-test with two tails was used to compare statistical difference between two groups. Results were presented as mean ± standard error of mean (SEM) and differences were considered significant at a *P* value less than 0.05.

## Results

### IL-29 plays an antiviral role in response to H1N1 virus infection

**Figure 1:**
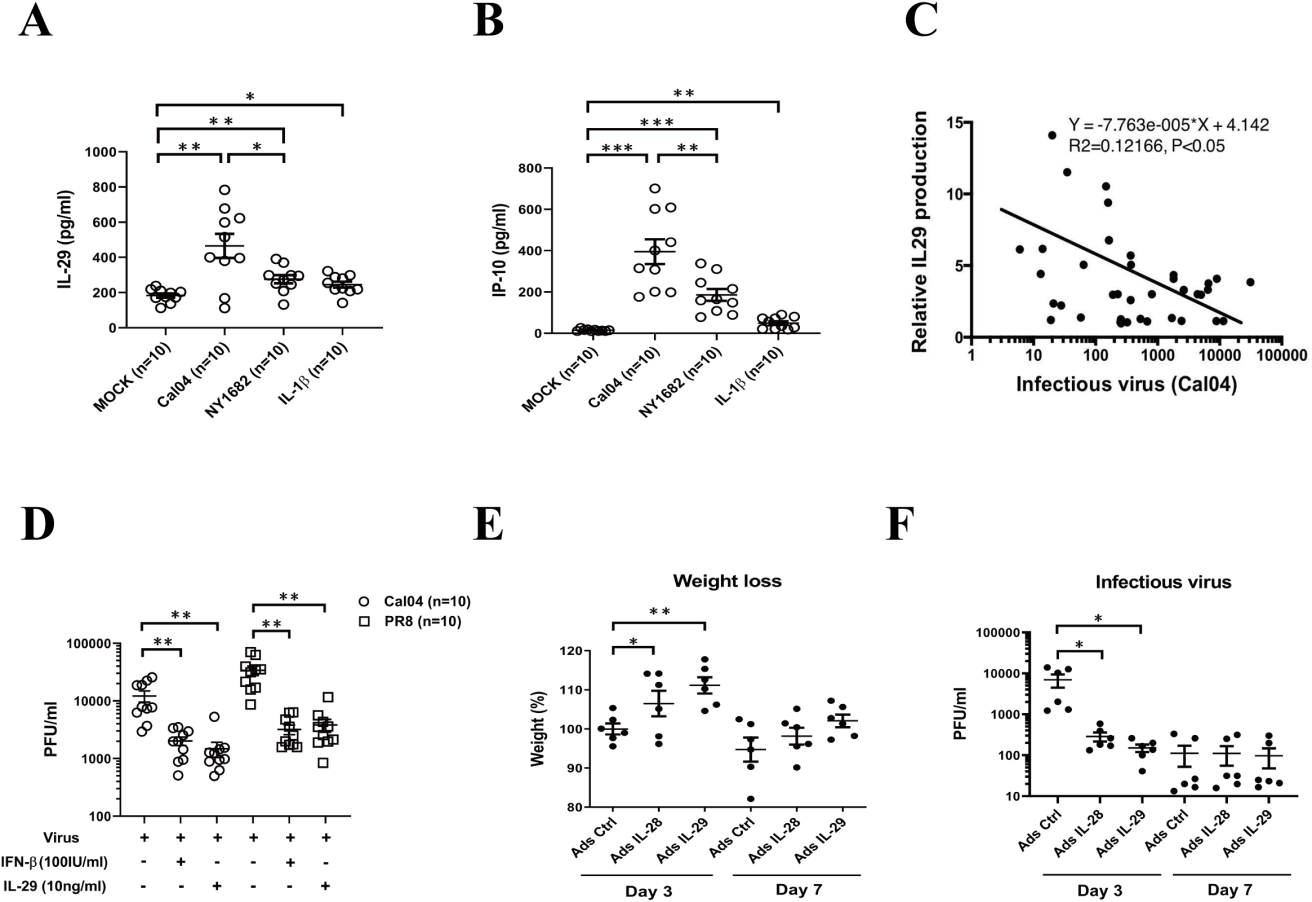
IL-29 exerts antiviral role against H1N1 virus infections. **(A, B)**, human AECs from the same donors (n=10) were infected with and without Cal04 or NY1682 (MOI=1) or treated with recombinant human IL-1β (10 ng/ml) as a positive control, and culture supernatant was collected 24 hours after viral infection for detection of IL-29 and IP-10 by ELISA. **(C)** Negative correlation between IL-29 expression in AECs and infectious virus release in AECs. IL-29 secretion was measured by ELISA and infectious virus release was evaluated by plaque assay. **(D)** IL-29 and IFN-β treatment directly reduced viral replication in human AECs in response to H1N1 infection. Human AECs from the same donors (n=10) were infected with Cal04 or PR8 (MOI=1) and treated with or without IFN-β or IL-29, and culture supernatant was collected 24 hours after viral infection for detection of virus release by plaque assay. **(E)** Wild-type C57BL/6 mice were inoculated with either an empty adenovirus vector or adenovirus overexpressing IL-29 or IL-28, followed by 100 pfu of PR8 virus challenge. Mice weight was monitored on day 3 or day 7 after PR8 virus challenge. **(F)** Wild-type C57BL/6 mice were inoculated with either an empty adenovirus vector or adenovirus overexpressing IL-29 or IL-28, followed by 100 pfu of PR8 virus challenge. The BAL fluid were collected on day 3 and day 7 and plaque assays were performed to detect viral burden. Significant differences are indicated as follows: *p<0.05, **p<0.01, and***p<0.001.

Human AECs serve as the first lines of pathogen defense in lung tissue. Previously, we have reported that as the direct targets for H1N1 virus, human AECs showed different susceptibility to H1N1 pdm09 virus strains Cal04 and NY1682, with a lower susceptibility to the Cal04 strain (28). Given the important roles of type III IFNs in antiviral defense of the mucosal barriers, we decided to explore whether the difference in infectivity of H1N1 pdm09 viruses NY1682 and Cal04 in AECs is related to the secreted type III IFNs. We quantified IL-29 (the only IFN-λ secreted by AECs) and IP-10 (the IL-29-induced ISG molecule associated with viral clearance) by ELISA and found that AECs secreted much greater level of IL-29 and IP-10 in response to Cal04 stimulation compared to NY1682 infection or IL-1β treatment, suggesting that the higher level of IL-29 might correlate to the lower infection rate of Cal04 in AECs (**Figures 1A, B**). Considering that age and single nucleotide polymorphisms of IFNL may affect the secretion of IL-29, the ten AEC cells we selected were all from adult donors (18-60 years old) and their genotype distribution of the rs12979860 (IL-28 C/T) or rs8099917 (IL-28 T/G) was consistent with the distribution pattern in the population (**Supplementary Table 1**).

To further investigate whether the lower infectivity of H1N1 virus was associated with higher IL-29 secretion, we performed a correlation analysis between Cal04 viral replication and IL-29 production. A significant negative correlation was found between viral load and the level of IL-29 production in Cal04-infected AECs, suggesting that the lower infectivity and replication capacity of Cal04 in AECs is related to higher secretion of IL-29 (**Figure 1C**). The 37 AEC cells we selected were all from adult donors, and their genotypic distribution was consistent with the distribution pattern in the population (**Supplementary Table 2**).

To assess the direct effect of IL-29 on H1N1 virus replication, we treated human AEC cells infected with H1N1 virus (Cal04 or PR8) *in vitro* with IFN-β or IL-29, and then detected the infectious viral release by plaque assay. As shown in **Figure 1D**, treatment with IL-29 or IFN-β (positive control) significantly reduced viral replication and release of infectious virus. The ten AEC cells we selected were all from adult donors and their genotype distribution was consistent with the distribution pattern in the population (**Supplementary Table 1**).

To further examine the direct effects of type III IFNs, particularly IL-29, on H1N1 virus replication, we treated mice with either an empty adenovirus vector or adenovirus expressing IL-29 or IL-28, followed by a PR8 virus challenge. Experiments in mice showed similar results. Compared with control mice, IL-29 or IL-28 overexpressing mice had a mild weight gain and a significant decrease in viral titer on day 3 after PR8 virus challenge. On day 7, PR8 viral titer dropped dramatically, probably due to the adaptive immune responses. And we did not observe significant differences in body weight and viral burden between control and IL-29 or IL-28 overexpressed groups on day 7 (**Figures 1E, F**). Together, these data suggest that IL-29 plays an antiviral role in response to H1N1 viral infection, especially during early stage of infection.

### IL-28B deficiency increases pulmonary inflammation in bacterial superinfections, but does not alter body weight or bacterial clearance

**Figure 2:**
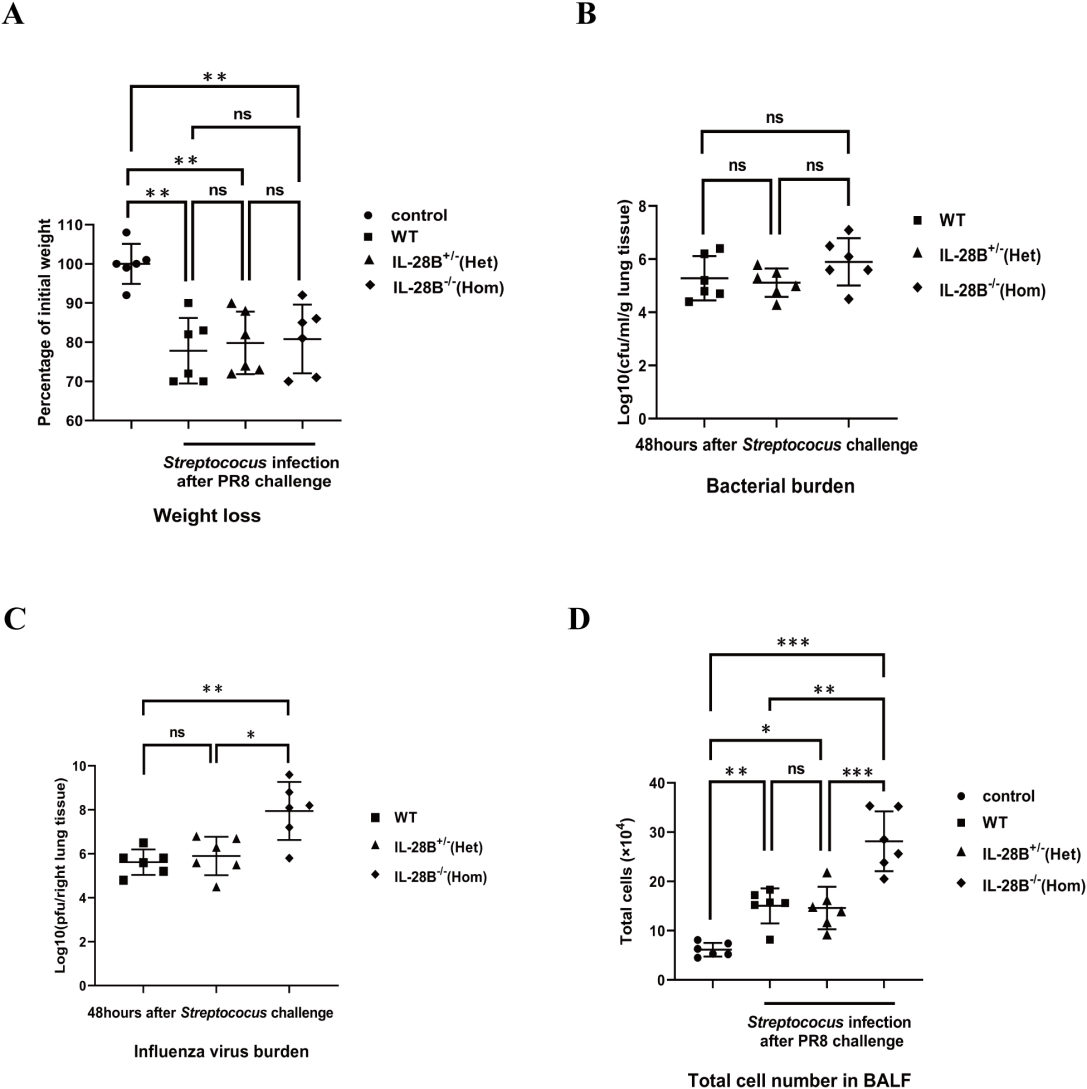
IL-28B deficiency increases pulmonary inflammation in bacterial superinfections, but does not alter body weight or bacterial clearance. Wild-type C57BL/6 mice, heterozygous IL-28B^+/-^ mice and homozygous IL-28B^-/-^ mice were respectively infected with 100 pfu PR8 virus and then challenged with 5×10^7^ CFU *Streptococus pneumoniae* per mouse and harvested 48 hours later. **(A)** Mice weight was monitored at 48 hours after bacterial challenge. **(B)** The BAL fluid were collected at 48 hours after bacterial challenge for assays of bacterial burden. **(C)** The right lungs were collected at 48 hours after bacterial challenge for assays of virus burden. **(D)** The BAL fluid were collected and the infiltrated cells in BALF was enumerated by a hemocytometer at 48 hours after bacterial challenge. Significant differences from the different group are indicated as follows: *p<0.05, **p<0.01, and ***p<0.001. ns, no significant difference.

*Streptococcus pneumoniae* is considered the “classic” pathogen found in influenza superinfection, a common complication of influenza. In addition to a role similar to that of IL-29 in direct lung defense against H1N1 influenza viruses, the role of IL-28B, another type III interferon, in the regulation of influenza/bacterial super-infection has been increasingly reported and emphasized (30, 31). To investigate the effect of IL-28B on bacterial superinfection, experiments were performed using IL-28B knockout mice (heterozygous IL-28B^+/-^ mice or homozygous IL-28B^-/-^ mice). The results showed that there were no differences in body weight and lung bacterial burden of mice between wild-type C57BL/6 mice, heterozygous IL-28B^+/-^ mice or homozygous IL-28B^-/-^ mice groups after PR8/*Streptococcus* challenge (**Figures 2A, B**). Consistently, IL-28B knockout mice (homozygous IL-28B^-/-^ mice) have slightly increased influenza virus burden in lung (**Figure 2C**). Notably, as a measurement of pulmonary inflammation, the count of infiltrated cells in bronchoalveolar lavage fluid (BALF) were significantly higher in the homozygous IL-28B^-/-^ mice groups than in other groups (**Figure 2D**). Taken together, our results showed that IL-28B deficiency increases pulmonary inflammation in bacterial superinfections and has no effect on body weight or bacterial clearance.

### Human AECs from young donors support a higher H1N1 replication and are not responsive to antiviral treatment

**Figure 3:**
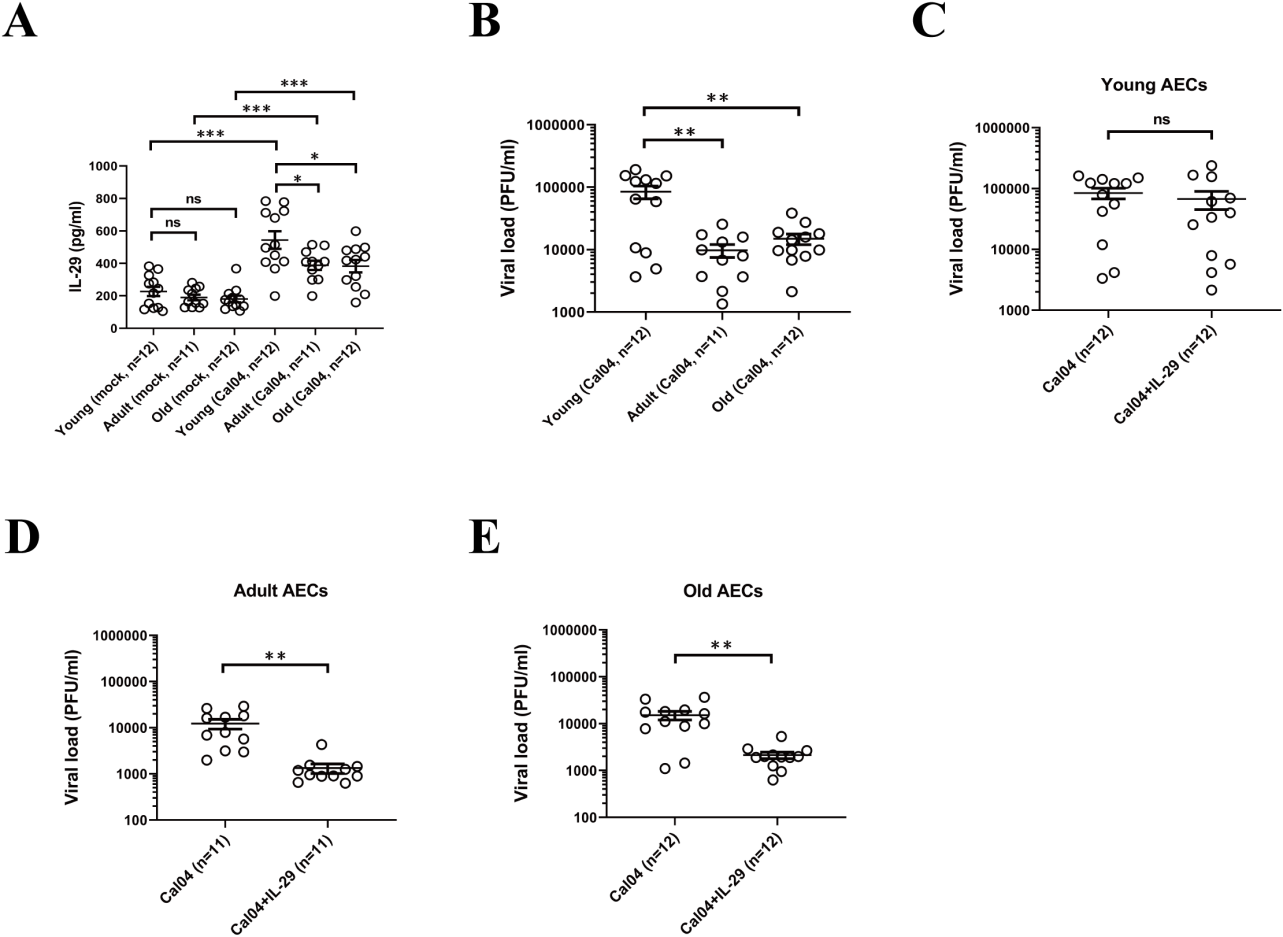
Human AECs from young donors support a higher H1N1 replication and are not responsive to antiviral treatment. **(A)** IL-29 expression level in Cal04 viruses-infected (MOI=1) and mock-infected AECs isolated from young, adult or old donors. The culture supernatants were collected 24 hours after viral infection for IL-29 detection by ELISA. **(B)** Virus titer in Cal04 viruses-infected (MOI=1) AECs isolated from young, adult or old donors. The culture supernatants were collected 24 hours after viral infection for virus titer detection by plaque assay. **(C)** Virus titer in Cal04 viruses-infected (MOI=1) AECs isolated from young donors with or without IL-29 treatment. The supernatants of AECs treated with or without IL-29 were collected 24 hours after viral infection for virus titer detection by plaque assay. **(D)** Virus titer detected by plaque assay in Cal04 viruses-infected (MOI=1) AECs isolated from adult donors with or without IL-29 treatment. **(E)** Virus titer detected by plaque assay in Cal04 viruses-infected (MOI=1) AECs isolated from old donors with or without IL-29 treatment. The genotype distribution of AECs among rs12979860 and rs8099917 was consistent with the distribution pattern in the population and matched in youth, adult and old groups. Significant differences are indicated as follows: *p<0.05, **p<0.01, and ***p<0.001. ns, no significant difference.

Human AECs from different donors are differentially susceptible to H1N1pdm09 viruses (28). To test whether the differences in H1N1 virus infectivity, replication capacity and susceptibility to antiviral strategies are partly due to age differences (younger donors <18 years old, adult donors in 18-60 years old and older donors >60 years old was specified). we inoculated primary AECs isolated from donors with different age with H1N1pdm09 Cal04 viruses or lab-adapted H1N1 PR8 viruses at a multiplicity of infection (MOI) of 1. Considering that single nucleotide polymorphisms in IFNL may also affect the infectivity, replication capacity and susceptibility to antiviral strategies of H1N1 virus, we matched the genotype and allele frequency distribution in youth, adult and elderly groups (**Supplementary Table 3**). Firstly, by using ELISA, we detected that AECs isolated from young donors in response to Cal04 viruses (or PR8 viruses) infection produced higher levels of IL-29 than both adult and old donors (**Figure 3A**, **Supplementary Figure 1A**). Surprisingly, despite of the higher level of IL-29, the viral titers in AECs from young donors were much higher than those in AECs from adult or old donors (**Figure 3B**, **Supplementary Figure 1B**). In addition, treatment with IL-29 did not suppress H1N1 viral load in AECs from young donors (**Figure 3C**, **Supplementary Figure 1C**), whereas AECs from adult and older donors were more sensitive to anti-H1N1 virus treatment with IL-29 (**Figures 3D, E**, **Supplementary Figures 1D, E**). These results suggest that although AECs from young donors produce more IL-29 after Cal04 or PR8 infection, they support a higher H1N1 replication and do not respond to -antiviral treatment with IL-29.

### The effects of rs12979860 or rs8099917 polymorphisms on replication of H1N1 virus and antiviral treatment by IL-29

**Figure 4:**
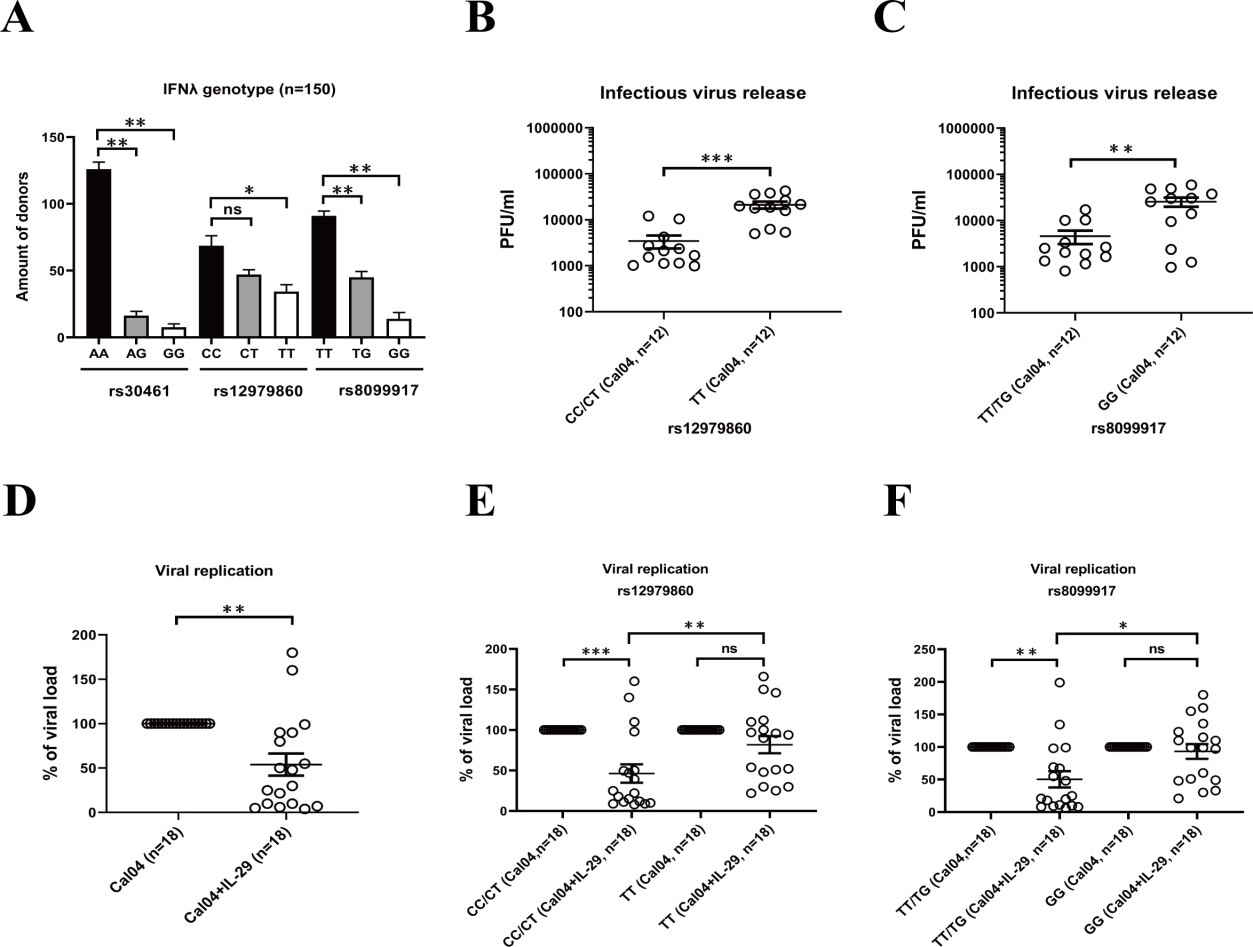
The effects of rs12979860 or rs8099917 polymorphisms on replication of H1N1 virus and antiviral treatment by IL-29. **(A)** Distribution of IL-28B/IL-29 SNPs in lung donors. The genotype distribution of the rs12979860 (IL-28 C/T), rs8099917 (IL-28 T/G) and rs30461 (IL-29 A/G) polymorphisms in 150 lung donors was analyzed by DNA extraction and genotyping. **(B)** The effect of rs12979860 polymorphism on replication of H1N1 virus. AECs isolated from adult donors with different genotypes in rs12979860 polymorphism were infected with H1N1 virus Cal04 at an MOI of 1. Supernatants were collected 24 hours after viral infection for detection of virus release by plaque assay. **(C)** The effect of rs8099917 polymorphism on replication of H1N1 virus. AECs isolated from adult donors with different genotypes in rs8099917 polymorphism were infected with H1N1 virus Cal04 at an MOI of 1. Virus release was detected by plaque assay. **(D)** Antiviral effect of IL-29 against H1N1 virus. The human AECs isolated from adult donors were infected with H1N1 virus Cal04 at an MOI of 1 and treated with or without IL-29. The supernatants of AECs were collected 24 hours after viral infection for detection of viral replication by plaque assay. **(E)** The effect of rs12979860 polymorphism on antiviral effect of IL-29 against H1N1 virus. The human AECs isolated from adult donors with rs12979860 CC/CT or rs12979860 TT were infected with H1N1 virus Cal04 at an MOI of 1 and treated with or without IL-29. Viral replication was detected by plaque assay. **(F)** The effect of rs8099917 polymorphism on antiviral effect of IL-29 against H1N1 virus. The human AECs isolated from adult donors with rs8099917 TT/TG or rs8099917 GG were infected with H1N1 virus Cal04 at an MOI of 1 and treated with or without IL-29. Viral replication was detected by plaque assay. Significant differences are indicated as follows: *p<0.05, **p<0.01, and ***p<0.001. ns, no significant difference.

Single nucleotide polymorphisms (SNPs) within the IFNL genes determines HCV infection outcome and patient responsiveness to IFN therapy (21). To investigate the correlation of SNPs in the IFNL gene with H1N1 influenza virus infection and outcomes, we firstly test the genotype distribution of the rs12979860 (IL-28 C/T), rs8099917 (IL-28 T/G) and rs30461 (IL-29 A/G) polymorphisms in 150 lung donors. We found that at rs30461 locus, AA is the major-allele, while AG or GG are minor-allele genotypes. At rs12979860 locus, CC is the major-allele, while CT or TT are minor-allele genotypes. And at rs8099917, TT is the major-allele and TG or GG are minor-allele genotypes (**Figure 4A**).

Firstly, we explored the association of rs12979860 (IL-28 C/T) polymorphism and rs8099917 (IL-28 T/G) polymorphism with the replication capacity of H1N1 pdm09 virus Cal04 or lab-adapted H1N1 virus PR8 in AECs. We selected AECs from adult donors and, in addition, when we analyzed the effect of rs12979860 (IL-28 C/T) polymorphism on Cal04 or PR8 virus replication, we matched rs8099917 (IL-28 T/G) polymorphism distribution in different groups and vice versa (**Supplementary Tables 4**, **5**). We inoculated human primary AECs isolated from donors with different genotypes in rs12979860 polymorphism or rs8099917 polymorphism with H1N1 virus Cal04 or PR8 at a MOI of 1 and assessed virus replication by plaque assay. The results showed that AECs with TT genotype in the rs12979860 polymorphism or the GG genotype in the rs8099917 polymorphism were more susceptible to H1N1 infection and supported more robust viral replication (**Figures 4B, C**, **Supplementary Figures 2A, B**).

Previous studies have shown that polymorphisms in the type III interferon gene affect the responsiveness of HCV viruses to antiviral treatment (21, 22). To investigate whether the rs12979860 (IL-28 C/T) polymorphism or rs8099917 (IL-28 T/G) polymorphism affects the antiviral effect of IL-29 against H1N1 virus, we infected AECs isolated from adult donors with the rs12979860 polymorphism or rs8099917 polymorphism with Cal04 or PR8 and treated them with IL-29, and then detected viral replication by plaque assay. First, consistent with the previous results, we found that IL-29 treatment significantly inhibited the replication of Cal04 or PR8 virus in AECs (**Figure 4D** and **Supplementary Figure 2C**). The genotype and allele frequency distribution in **Figure 4D**, **Supplementary Figure 2C** was shown in **Supplementary Table 6**. Furthermore, viral replication in AECs from the donors of rs12979860 CC/CT was significantly inhibited by the addition of IL-29, yet the viral load of AECs from the donors of rs12979860 TT was not inhibited and was significantly higher than that of rs12979860 CC/CT (**Figure 4E**, **Supplementary Figure 2D**). A similar trend was observed for rs8099917. Viral replication of AECs from the donors of rs8099917 TT/TG was significantly inhibited by the IL-29 treatment, whereas the viral load of AECs from the donors of rs8099917 GG was not inhibited and was significantly higher than that of rs8099917 TT/TG (**Figure 4F**, **Supplementary Figure 2E**). The AECs we selected were all from adult donors. In addition, when we analyzed the effect of rs12979860 (IL-28 C/T) polymorphism on IL-29 treatment, we matched rs8099917 (IL-28 T/G) polymorphism distribution in different groups and vice versa (**Supplementary Tables 7**, **8**). These results suggest that the rs12979860 polymorphism or the rs8099917 polymorphism affects virus replication in AECs and the antiviral effect of IL-29. It is likely that donors with rs12979860TT and rs8099917GG may respond poorly to -antiviral therapy with IL-29 and have a worse prognosis.

### AECs from donors with rs12979860 TT produce lower level of IFN in response to H1N1 infection

**Figure 5:**
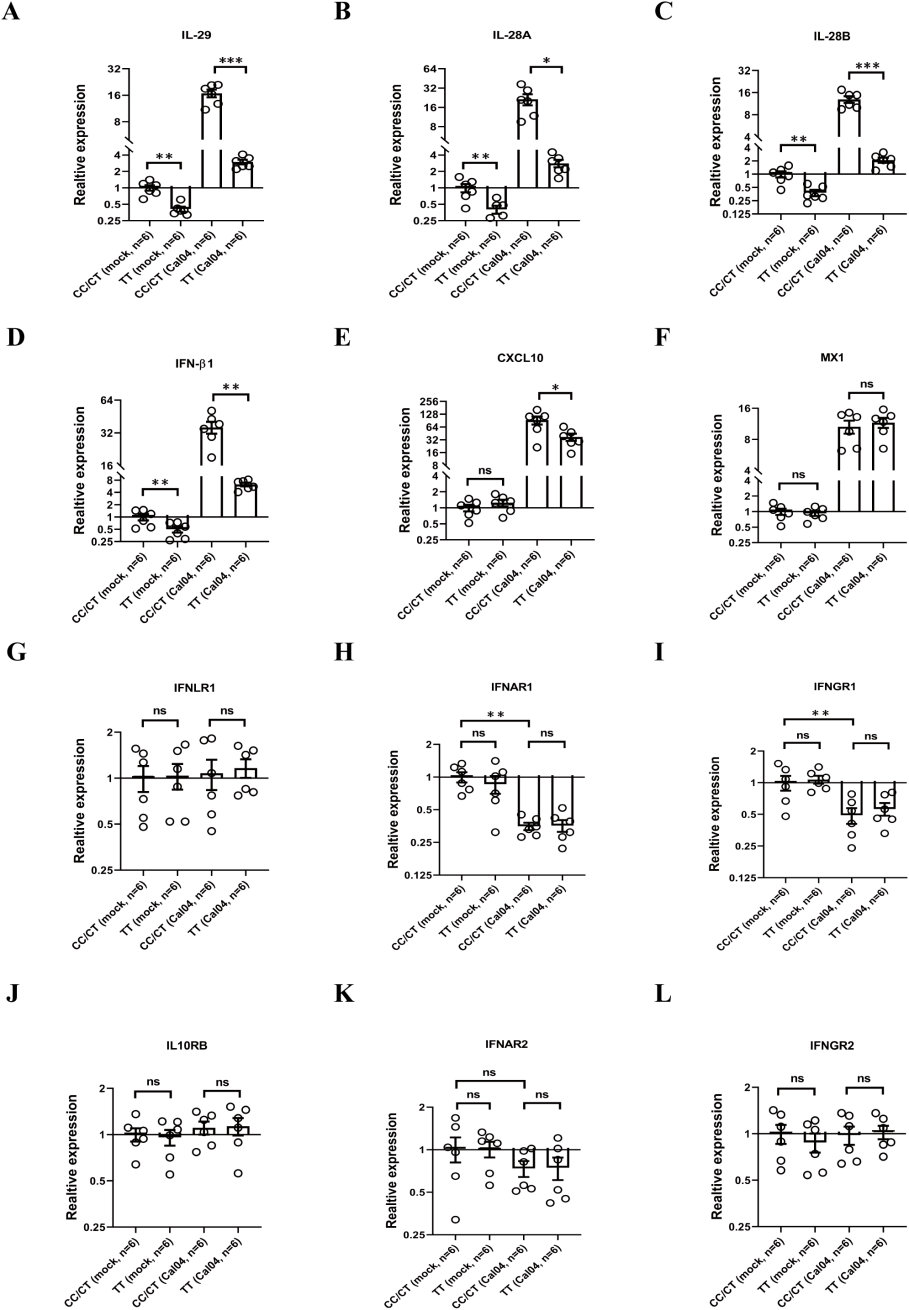
AECs from donors with rs12979860 TT produce less level of IFN in response to H1N1 infection, whereas rs12979860 polymorphism does not affect expression of IFN receptors. **(A–F)** AECs from donors with rs12979860 CC/CT or rs12979860 TT were infected with Cal04 virus at a MOI of 1 and the mRNAs expression of interferons and ISGs were examined by real-time PCR. **(G–L)** AECs from donors with rs12979860 CC/CT or rs12979860 TT were infected with Cal04 virus at a MOI of 1 and the mRNAs expression of IFN receptors was measured by real-time PCR. All AECs are collected from adult donors and their genotype distribution in rs8099917 was matched between different groups. Significant differences are indicated as follows: *p<0.05, **p<0.01, and ***p<0.001. ns, no significant difference.

Interferon is an important antiviral cytokine in influenza virus infection, even though it has side effects of exacerbating inflammation and impairing lung epithelial cell barriers and repair (32–34). Since the rs12979860 T/C polymorphism is located upstream of the promoter regions of the IL-28B gene as well as IL-28A and IL-29 genes and could in principle affect all three IFNL genes (29), we determined whether rs12979860 (IL-28 C/T) polymorphism alters interferon production. Firstly, we evaluated the gene expression of IFNs by real-time PCR. We found that human AECs from donors of rs12979860 TT produced lower mRNA levels of IL-29, IL-28A, IL-28B and IFNβ1 in response to Cal04 or PR8 virus infection or when resting uninfected (**Figures 5A–D**, **Supplementary Figures 3A–D**). In addition, the rs12979860 (IL-28 C/T) polymorphism affected Cal04 or PR8-induced expression of the IL-29-stimulated gene CXCL10, but not of the other IFN-stimulated gene (ISGs) Mx1 (**Figures 5E, F**, **Supplementary Figures 3E, F**). All AECs are collected from adult donors and their genotype distribution in rs8099917 are matched between groups (**Supplementary Table 9**). These results indicate that AECs from donors with different genotypes of the rs12979860 polymorphism display different IFN responses upon infection by H1N1 virus. Fewer IFN production in AECs from donors with rs12979860 TT may be associated with reduced viral clearance and poorer disease outcomes in individuals.

Next, we investigated whether the rs12979860 (IL-28 C/T) polymorphism affects the expression of IFN receptors in AECs in response to H1N1 virus infection. We inoculated primary AECs isolated from donors with different genotypes in rs12979860 polymorphism with H1N1 virus Cal04 or PR8 at a MOI of 1 and evaluated the expression of IFN receptors by real-time PCR. All AECs are collected from adult donors and their genotype distribution in rs8099917 are matched between groups (**Supplementary Table 9**). The IFN receptors examined in this study include: type I interferon receptors (interferon α and β receptor subunits 1 and 2, IFNAR1 and IFNAR2), type II interferon receptors (interferon γ receptors 1 and 2, IFNGR1 and IFNGR2), type III interferon receptors (interferon λ receptor 1, IFNLR1 and interleukin 10 receptor subunit beta [IL-10RB]). The results showed that although the expression of IFNAR1, IFNAR2 or IFNGR1 was down-regulated upon infection with Cal04 or PR8 viruses, there was no statistical difference in the expression levels of these three IFN receptors between AECs with CC/CT and TT genotypes, either in the absence of infection or after viral infection (**Figures 5G–L**, **Supplementary Figures 3G–L**), indicating that rs12979860 polymorphism does not affect expression of IFN receptors in AECs.

### Rs12979860 genotype impairs IL-29 promoter activity

**Figure 6:**
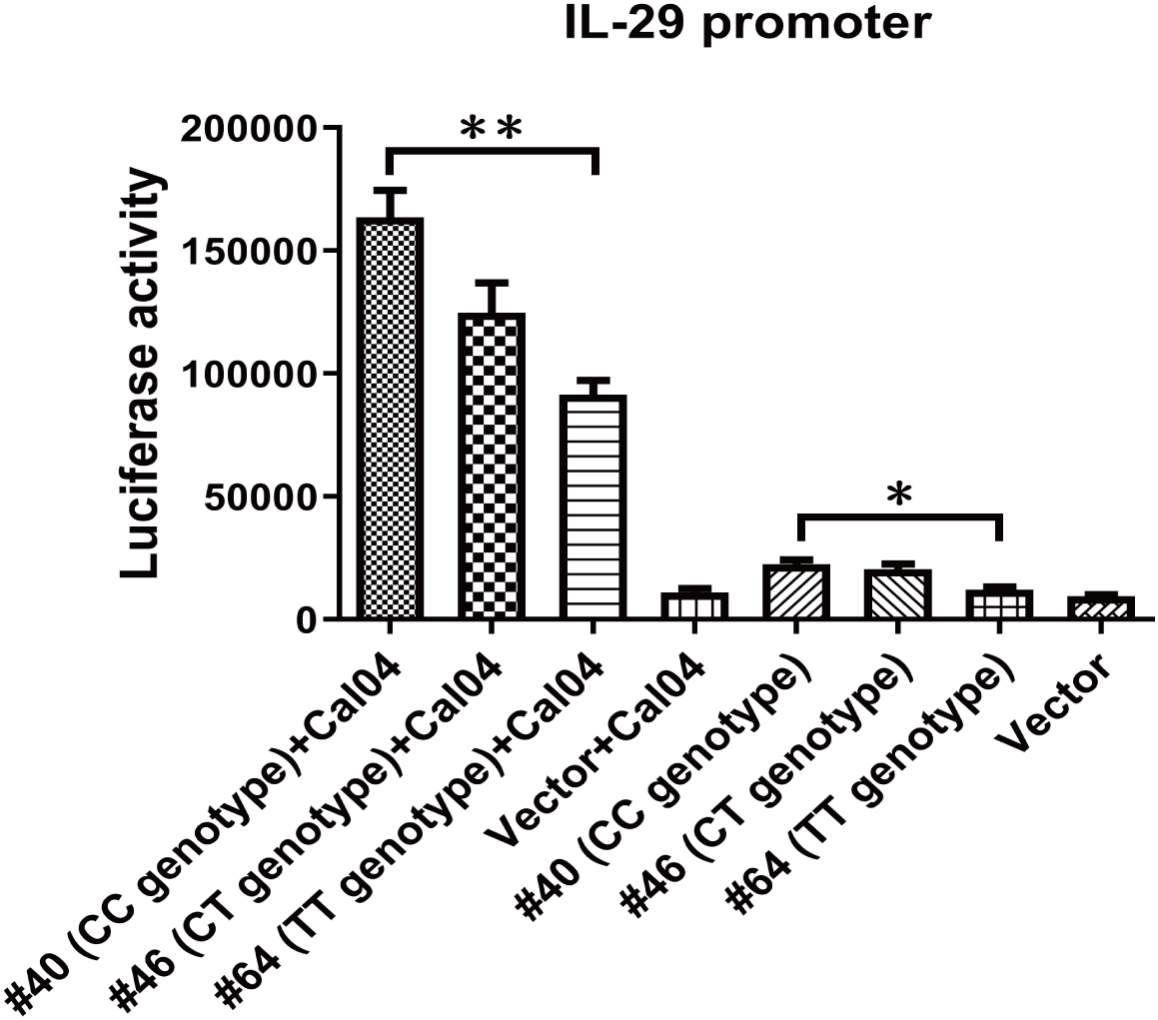
Rs12979860 genotype determines IL-29 promoter activity. IL-29’s promoter was amplified from adult donor 40 with rs12979860 CC (#40), or adult donor 46 with rs12979860 CT(#46) or adult donor 64 with rs12979860 TT (#64) and constructed into pGL3 basic vector. The A549 cells were transfected with these constructs and then challenged with Cal04 virus. The differences in promoter activity of IL-29 due to genotype of rs12979860 were measured using a dual-luciferase reporter assay. Firefly luciferase expression was normalized to Renilla luciferase expression. Significant differences are indicated as follows: *p<0.05, **p<0.01.

The rs12979860 T/C polymorphism is located upstream of the promoter region of the IL-28B gene as well as the IL-28A and IL-29 genes and could in principle affect all three IFN-lambda genes (29). In addition, the results in our previous section demonstrated that IL-29 exerts a direct antiviral effect in response to H1N1 virus infection and that the rs12979860 polymorphism affects IL-29 expression. Thus, we proceeded to determine if rs12979860 could directly alter IL-29 promoter activity. Three reporter constructs were created with the IL-29 core promoter that drives the expression of luciferase, one with the CC genotype from donor #40, one with the CT genotype from donor #46, and the other with the TT genotype from donor #64, and then were transfected into A549 cells separately. The results showed that the promoter of the TT genotype from donor 64# exhibited significantly reduced activity, both in the resting uninfected state and in Cal04 or PR8 virus-stimulated cells, compared to the group from donor #40 (**Figure 6**, **Supplementary Figure 4**). Above data indicate that individuals carrying the rs12979860 TT genotype possess lower promoter activity of IL-29 upon H1N1 virus infection, correlating with the reduced expression levels of IL-29.

### Increased allele frequency of rs12979860 T and rs8099917 G correlates with increase of BMI

**Figure 7:**
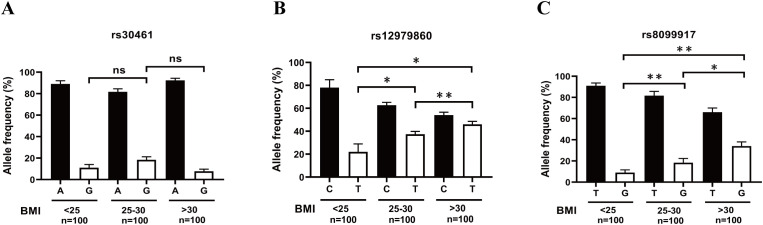
Increased allele frequency of rs12979860 T and rs8099917 G correlates with increased BMI. **(A)** The correlation between the allele frequency of rs30461A or G and BMI was analyzed by genotyping and BMI analysis. **(B)** The correlation between the allele frequency of rs12979860 C or T and BMI was analyzed by genotyping and BMI analysis. **(C)** The correlation between the allele frequency of rs8099917 T or G and BMI was analyzed by genotyping and BMI analysis. Significant differences are indicated as follows: *p<0.05, **p<0.01. ns, no significant difference.

A previous study demonstrated that AECs of obese subjects were more likely to be infected with H1N1pdm09 than those of non-obese subjects (28). Therefore, we further explored the association of rs12979860 polymorphism or the rs8099917 polymorphism with obesity. After genotyping and BMI analysis, we found that increased allele frequencies of rs12979860 T and rs8099917 G were associated with an increase in BMI (**Figures 7B, C**). However, there was no correlation between the allele frequency of rs30461 and BMI (**Figure 7A**). These results suggest that individuals carrying the rs12979860 allele T and the rs8099917 allele G with high BMI may be more susceptible to H1N1 infection.

## Discussion

Host factors, such as innate immune response, genetic polymorphisms, age, and body weight might be the important determinants of susceptibility, severity, and responsiveness to treatment of influenza disease and are attracting increasing attention. Our study here provides valuable insights into the host determinants of H1N1 outcomes, highlighting IFN-λ polymorphisms (rs12979860, rs8099917) as key modulators of antiviral responses and age-dependent treatment resistance. IL-28 and IL-29 exhibit dual antiviral and anti-inflammatory roles, with genetic variants impairing IFN production and therapeutic efficacy.

Respiratory epithelial cells are the first line of defense against respiratory virus invasion through their innate immune system, which is mediated by increased secretion of IFN, a key antiviral cytokine (35, 36). Type I IFNs (predominantly IFN-α and IFN-β) represent a classical way for host in combating viral infection (37, 38). Type III IFNs (IFN-λ family: IL-28A, IL-28B, IL-29, and IFN-λ4) is a class of IFNs with antiviral characteristics similar to those of IFN-α and IFN-β, activating the same JAK/STAT signal transduction cascade for transcriptional activation of ISGs (39, 40). However, mouse models of influenza A virus infection have shown that treatment with IFN-α leads to a transient upsurge in ISG expression in primary AECs, whereas IFN-λ treatment leads to a long-term induction of ISG in primary AECs and a sustained antiviral protection in both lower and upper respiratory tract *in vivo* (41). Furthermore, IFN-λ is critical for barrier integrity in the epithelial cells that are frequently attacked by virus, while the type I interferon ramps up at these sites only after the epithelial cell barrier fails to constrain the virus (42). Besides, IL-29 (IFN-λ1) is the major IFN protein secreted by alveolar type II epithelial cells, and differentiated ATIIs is the main source of IL-29 production during influenza A virus infection (43). IL-29 treatment induced a dose-dependent increase of antiviral gene expression and a decrease of infectious virus release and virus-induced cytokine response (43). Consistent with this report, our results found that, IL-29 treatment directly inhibited the replication of H1N1 virus *in vitro* and was comparable to the antiviral effect of IFN-β (**Figure 1D**). *In vivo*, IL-28 and IL-29 overexpressing mice also exhibit significantly inhibition of H1N1 virus replication (**Figures 1E, F**). These data suggest that IL-28 and IL-29 plays an antiviral role in response to H1N1 virus infection. Furthermore, because IFNAR is widely expressed in immune cells, IFN-α/β responses can lead to immunopathology during viral infection. The IFN-λ receptor (IFNLR) is mainly expressed in epithelial cells, thus restricting IFNλ responses in epithelial cells and eliminating the destructive pro-inflammatory effect that were often observed during IFN-α/β responses. Therefore, side effects such as high fever are much less frequent during IFN-λ therapy.

Human AECs are the primary target of pandemic H1N1 virus (28). Our previous studies have developed a primary culture system to study human AECs (24) and reported their innate immune response to influenza virus infection (27, 43). By using this system, Wang et al. investigated the infection of AECs with different H1N1pdm09 viruses Cal04 and NY1682, and found that Cal04 and NY1682, despite nucleotide similarity, showed divergent susceptibility in AECs (28). Because IL-29 is the only IFN-λ secreted by AECs infected with influenza A virus (IAV), and IP-10 is an IL-29-induced ISG molecule associated with viral clearance (43), we explored whether the divergent susceptibility in infection of different H1N1 viruses was due to the different secretion of IL-29 and IP-10. Our results showed that Cal04 viruses stimulated more production of IL-29 and IP-10 than NY1682 viruses or IL-1β (**Figures 1A, B**) and its replication capacity was negatively correlated with IL-29 production (**Figure 1C**). Our results might explain why Cal04 has a lower infection rate and poses less damage to the epithelial barrier in AECs.

Bacterial superinfections are often a complication of influenza and increase morbidity and mortality. Although IFN-λ has recently been proposed for the treatment of influenza infection and possesses many advantages, cautions should be exercised as it has been shown to impair bacterial clearance during influenza superinfection (30). In our work using IL-28 knockout mice, we found that IL-28 deficiency increased the number of infiltrating inflammatory cells in bronchoalveolar lavage fluid (BALF) and the load of H1N1 virus, but did not affect bacterial clearance (**Figure 2**), which supports the work by Rich et al. showing that IFN-λ can reduce neutrophil accumulation in BALF in influenza/MRSA superinfection (30). The effectiveness and safety of IFN-λ as a therapeutic agent for influenza needs to be assessed by further studies.

Age is a key determinant of influenza infection outcomes, with more severe effects typically observed in children and older adults. Hospitalization rates for children under one year old are comparable to those of older adults (44). Children also have the highest rate of symptomatic infection (45), along with longer viral duration and higher viral titers than adults (44). Consequently, children are considered to be human reservoirs of influenza infection and may play an important role in the spread of the virus throughout the community (46). Notably, a higher proportion of all influenza-related deaths occurs in children than in adults (47). Moreover, aging increases susceptibility to influenza viruses due to dysregulation of the immune system in the elderly. In the United States, people over 65 years of age bear the highest disease burden and substantial economic costs due to influenza. Ninety percent of people who died from seasonal flu are over age of 65 (48). A report on influenza-related excess mortality in Korea also showed that the mortality impact of influenza was particularly higher among people aged 65 years or older (49). However, the effect of age differences on the infection characteristics of H1N1 in AECs remains unclear. Consistent with previous reports, our study showed that although AECs from young donors produced more IL-29 after infection with H1N1 epidemic strain Cal04 or lab-adapted H1N1 strain PR8 than AECs from adult or older donors, they still supported higher H1N1 replication and weak responsiveness to antiviral treatment with IL-29 (**Figure 3**, **Supplementary Figure 1**). The detailed mechanisms need to be further investigated in the future.

Recent studies have highlighted specific single nucleotide polymorphisms (SNPs) in IFNL genes, such as rs12979860, rs8099917, and rs30461, which affect viral clearance, treatment responsiveness, and overall disease outcomes (21–23, 50, 51). However, whether these three SNPs may affect host immune responses to, or replication of, the A/H1N1 influenza virus remains unclear. In our present study, we investigated the association of these three SNPs with the viral replication, IL-29 antiviral treatment, IFN secretion, IFNR expression, IL-29 promoter activity in H1N1 virus infection in AECs. Our results showed that the polymorphism of rs12979860 (IL-28 C/T) and rs8099917 (IL-28 T/G) does affect the replication of H1N1 virus (**Figures 4B, C**, **Supplementary Figures 2A, B**) and the antiviral effects of IL-29 in AECs, the donors with rs12979860TT and rs8099917GG exhibit nonresponsiveness to IL-29 antiviral effects (**Figures 4E, F**, **Supplementary Figures 2D, E**). Controversial results exist in literatures regarding the effect of IL-28B polymorphisms on IFN-λ expression. Jin et al. suggested that the rs8099917G variant may lead to upregulation of IL-28B and ISG expression (52). However, Urban et al. did not found the relationship between the IL-28B genotype and IL-28B expression (53). Through analyzing the relationship between rs12979860 polymorphism and IFN response, our results showed that rs12979860 TT variant produced lower levels of IL-29, IL-28A, IL-28B, IFNβ1 and CXCL10 (ISGs of IL-29) in the absence of virus infection or in response to Cal04 or PR8 virus infection, while having no effect on the expression of ISGs-Mx1 (**Figures 5A–F**, **Supplementary Figures 3A–F**). Further investigation to the mechanism demonstrated that individuals carrying the rs12979860 TT genotype had reduced IL-29 promoter activity (**Figure 6** and **Supplementary Figure 4**). The relationship between IFN-λR expression and IFNL genotype polymorphisms has important implications for the antiviral therapy. Duong et al. showed increased expression of IFNλR1 in chronic hepatitis C patients carrying rs8099917G (54). However, our study did not find differences in the expression of all IFN receptors in AECs with rs12979860 CC/CT or rs12979860 TT genotypes in response to Cal04 or PR8 virus infection (**Figures 5G–L**, **Supplementary Figures 3G–L**). Besides that, Obese hosts are at high risk of contracting influenza and suffering serious sequelae after infection (55). We found that increased allele frequencies of rs12979860 T and rs8099917 G were correlated with increased BMI (**Figures 7B, C**). All these data imply that rs12979860 TT and rs8099917 GG may be an unfavorable genotype for host in response to H1N1 virus infection, with reduced viral clearance, reduced response to IL-29 treatment, and increased disease severity.

In conclusion, our study has investigated the impact of IFN-λ, age of the host, and SNPs of the IFNL gene on H1N1 virus infection and replication, innate immune response, and responsiveness to IL-29 treatment. To the best of our knowledge, this is the first study to investigate the relationship between H1N1 virus and IL-28B/IL-29 polymorphisms. Identification of genetic variants in the IFNL gene associated with H1N1 virus infection has the potential to improve the decision-making process for anti-influenza treatment. Our study highlighted the IFN-λ signaling axis as a potential therapeutic target for development of novel anti-influenza drugs.

## Data Availability

The original contributions presented in the study are included in the article/**Supplementary Material**. Further inquiries can be directed to the corresponding authors.

## Supplementary material

Supplementary Figure 1 Human AECs from young donors support higher H1N1 viral replication and do not respond to antiviral treatment with IL-29. **(A)** IL-29 expression level in PR8 viruses-infected (MOI=1) and mock-infected AECs isolated from young, adult or old donors. The culture supernatants were collected 24 hours after viral infection for IL-29 detection by ELISA. **(B)** Virus titer in PR8 viruses-infected (MOI=1) AECs isolated from young, adult or old donors. The culture supernatants were collected 24 hours after viral infection for virus titer detection by plaque assay. **(C)** Virus titer detected by plaque assay in PR8 viruses-infected (MOI=1) AECs isolated from young donors with or without IL-29 treatment. **(D)** Virus titer detected by plaque assay in PR8 viruses-infected (MOI=1) AECs isolated from adult donors with or without IL-29 treatment. **(E)** Virus titer detected by plaque assay in PR8 viruses-infected (MOI=1) AECs isolated from old donors with or without IL-29 treatment. . Significant differences are indicated as follows: *p<0.05, **p<0.01, and ***p<0.001. ns, no significant difference.

Supplementary Figure 2 The polymorphisms of rs12979860 or rs8099917 affect the replication of H1N1 virus and antiviral treatment by IL-29. **(A)** The effect of rs12979860 polymorphism on replication of H1N1 virus. AECs isolated from adult donors with different genotypes in rs12979860 polymorphism were infected with H1N1 virus PR8 at an MOI of 1. Supernatants were collected 24 hours after viral infection for detection of virus release by plaque assay. **(B)** The effect of rs8099917 polymorphism on replication of H1N1 virus. AECs isolated from adult donors with different genotypes in rs8099917 polymorphism were infected with H1N1 virus PR8 at an MOI of 1.Virus release was detected by plaque assay. **(C)** Antiviral effect of IL-29 against H1N1 virus. The human AECs isolated from adult donors were infected with H1N1 virus PR8 at an MOI of 1 and treated with or without IL-29. The supernatants of AECs were collected 24 hours after viral infection for detection of viral replication by plaque assay. **(D)** The effect of rs12979860 polymorphism on antiviral effect of IL-29 against H1N1 virus. The human AECs isolated from adult donors with rs12979860 CC/CT or rs12979860 TT were infected with H1N1 virus PR8 at an MOI of 1 and treated with or without IL-29. Viral replication was detected by plaque assay. **(E)** The effect of rs8099917 polymorphism on antiviral effect of IL-29 against H1N1 virus. The human AECs isolated from adult donors with rs8099917 TT/TG or rs8099917 GG were infected with H1N1 virus PR8 at an MOI of 1 and treated with or without IL-29.Viral replication was detected by plaque assay. Significant differences are indicated as follows: *p<0.05, **p<0.01, and ***p<0.001. ns, no significant difference.

Supplementary Figure 3 AECs from donors with rs12979860 TT produce less level of IFN in response to H1N1 infection, whereas rs12979860 polymorphism does not affect expression of IFN receptors. **(A–F)** AECs from donors with rs12979860 CC/CT or rs12979860 TT were infected with PR8 virus at a MOI of 1 and the mRNAs expression of interferons and ISGs were examined by real-time PCR. **(G–L)** AECs from donors with rs12979860 CC/CT or rs12979860 TT were infected with PR8 virus at a MOI of 1 and the mRNAs expression of IFN receptors was measured by real-time PCR. All AECs are collected from adult donors and their genotype distribution in rs8099917 was matched between different groups. Significant differences are indicated as follows: *p<0.05, **p<0.01, and ***p<0.001. ns, no significant difference.

Supplementary Figure 4 Rs12979860 genotype determines IL-29 promoter activity. IL-29’s promoter was amplified from adult donor 40 with rs12979860 CC (#40), or adult donor 46 with rs12979860 CT(#46) or adult donor 64 with rs12979860 TT (#64) and constructed into pGL3 basic vector. The A549 cells were transfected with these constructs and then challenged with PR8 virus. The differences in promoter activity of IL-29 due to genotype of rs12979860 were measured using a dual-luciferase reporter assay. Firefly luciferase expression was normalized to Renilla luciferase expression. Significant differences are indicated as follows: *p<0.05.
