# Supplementary material for: Type III interferon, age and IFNL gene single nucleotide polymorphisms determine the characteristics of H1N1 influenza infection
Source: Front Immunol. 2025 May 14;16:1592841. doi: 10.3389/fimmu.2025.1592841 (PMC12116514; doi:10.3389/fimmu.2025.1592841)
Supplement: Supplementary Figure 1 — Human AECs from young donors support higher H1N1 viral replication and do not respond to antiviral treatment with IL-29. (A) IL-29 expression level in PR8 viruses-infected (MOI=1) and mock-infected AECs isolated from young, adult or old donors. The culture supernatants were collected 24 hours after viral infection for IL-29 detection by ELISA. (B) Virus titer in PR8 viruses-infected (MOI=1) AECs isolated from young, adult or old donors. The culture supernatants were collected 24 hours after viral infection for virus titer detection by plaque assay. (C) Virus titer detected by plaque assay in PR8 viruses-infected (MOI=1) AECs isolated from young donors with or without IL-29 treatment. (D) Virus titer detected by plaque assay in PR8 viruses-infected (MOI=1) AECs isolated from adult donors with or without IL-29 treatment. (E) Virus titer detected by plaque assay in PR8 viruses-infected (MOI=1) AECs isolated from old donors with or without IL-29 treatment. . Significant differences are indicated as follows: *p<0.05, **p<0.01, and ***p<0.001. ns, no significant difference. [file DataSheet1.zip › 20250313 supplementary materials/20250313 Supplementary Table 3.doc]

Supplementary Table 3A

Comparison of genotype and allele frequency distribution among rs12979860 and rs8099917 of human AECs from young, adult or old donors in Figure 3 and Supplementary Figure 1.

| **Rs12979860** | **Rs8099917** | **Number of donors** | | |
| --- | --- | --- | --- | --- |
|  |  | Young (n=12) | Adult (n=11) | Old (n=12) |
| CC | TT | 4 | 4 | 4 |
| CC | TG | 2 | 2 | 2 |
| CT | TT | 3 | 2 | 3 |
| CT | TG | 1 | 1 | 1 |
| TT | TG | 1 | 1 | 1 |
| TT | GG | 1 | 1 | 1 |

Supplementary Table 3B

Comparison of genotype and allele frequency distribution among rs12979860 and rs8099917 of human AECs from young, adult or old donors in Figure 3 and Supplementary Figure 1.

| **SNP** | **Number of donors** | | | **P value** |
| --- | --- | --- | --- | --- |
|  | Young(n=12) | Adult(n=11) | Old(n=12) |  |
| **Rs12979860** |  | | |  |
| CC | 6(50%) | 6(55%) | 6(50%) | p>0.05 |
| CT | 4(33%) | 3(27%) | 4(33%) | p>0.05 |
| TT | 2(17%) | 2(18%) | 2(17%) | p>0.05 |
| **Rs8099917** |  | | |  |
| TT | 7(58%) | 6(55%) | 7(58%) | p>0.05 |
| TG | 4(33%) | 4(36%) | 4(33%) | p>0.05 |
| GG | 1(9%) | 1(9%) | 1(9%) | p>0.05 |

Note：SNP, Single nucleotide polymorphism

The genotype and allele frequency distribution among rs12979860 and rs8099917 was consistent with the distribution pattern in the population (see Figure 4A), and the genotype distributions were matched in young, adult and old groups.
